# CRISPR/Cas9-Mediated Targeted Mutagenesis of *FtMYB45* Promotes Flavonoid Biosynthesis in Tartary Buckwheat (*Fagopyrum tataricum*)

**DOI:** 10.3389/fpls.2022.879390

**Published:** 2022-05-12

**Authors:** Dong Wen, Lan Wu, Mengyue Wang, Wei Yang, Xingwen Wang, Wei Ma, Wei Sun, Shilin Chen, Li Xiang, Yuhua Shi

**Affiliations:** ^1^Key Laboratory of Beijing for Identification and Safety Evaluation of Chinese Medicine, Artemisinin Research Center, Institute of Chinese Materia Medica, China Academy of Chinese Medical Sciences, Beijing, China; ^2^College of Pharmaceutical Sciences, Heilongjiang University of Chinese Medicine, Harbin, China

**Keywords:** *Fagopyrum tataricum*, PTG/Cas9 system, targeted genome editing, *FtMYB45* gene, flavonoid biosynthesis

## Abstract

The clustered regularly interspaced short palindromic repeat/CRISPR-associated protein 9 (CRISPR/Cas9) technology is an efficient genome editing tool used in multiple plant species. However, it has not been applied to Tartary buckwheat (*Fagopyrum tataricum*), which is an important edible and medicinal crop rich in rutin and other flavonoids. *FtMYB45* is an R2R3-type MYB transcription factor that negatively regulates flavonoid biosynthesis in Tartary buckwheat. Here, the CRISPR/Cas9 system polycistronic tRNA-sgRNA (PTG)/Cas9 was employed to knock out the *FtMYB45* gene in Tartary buckwheat. Two single-guide RNAs (sgRNAs) were designed to target the second exon of the *FtMYB45* gene. Twelve transgenic hairy roots were obtained using *Agrobacterium rhizogenes-*mediated transformation. Sequencing data revealed that six lines containing six types of mutations at the predicted double-stranded break site were generated using sgRNA1. The mutation frequency reached 50%. A liquid chromatography coupled with triple quadrupole mass spectrometry (LC-QqQ-MS) based metabolomic analysis revealed that the content of rutin, catechin, and other flavonoids was increased in hairy root mutants compared with that of lines transformed with the empty vector. Thus, CRISPR/Cas9-mediated targeted mutagenesis of *FtMYB45* effectively increased the flavonoids content of Tartary buckwheat. This finding demonstrated that the CRISPR/Cas9 system is an efficient tool for precise genome editing in Tartary buckwheat and lays the foundation for gene function research and quality improvement in Tartary buckwheat.

## Introduction

Tartary buckwheat [*Fagopyrum tataricum* (L.) Gaertn], also called bitter buckwheat, is an annual dicotyledonous plant belonging to the Polygonaceae family and the Fagopyrum genus. It is a diploid species (2*n* = 2*x* = 16), originates in the mountains of Western China at 400–3,900m of altitude, and is mainly cultivated in the Himalayas, Southeast Asia, Europe, and South America, particularly in China, Afghanistan, Bhutan, Kazakhstan, Northern India, and Nepal (Wang and Campbell, 2007; Guo et al., 2011). Tartary buckwheat is an important traditional medicinal and edible plant, which is considered a new plant-based ingredient to enrich corn-based gluten-free formulations (Appiani et al., 2021). It is rich in various flavonoids, high-quality proteins, amino acids, and dietary fiber (Zhao et al., 2012). Flavonoids such as rutin, catechin, and epicatechin are the most important biologically active components of Tartary buckwheat (Md et al., 2013). Studies have shown that flavonoids effectively improve the symptoms of and prevent cardiovascular diseases, hypertension, diabetes, retinal hemorrhage, and acute hemorrhagic nephritis. They also have positive effects on the stomach, promote digestion, and improve immunity (Martínez Conesa et al., 2005; Jiang et al., 2007; Tomotake et al., 2007). Thus, Tartary buckwheat has become an important functional food (Zhou et al., 2015).

MYB transcription factors play important roles in the regulation of flavonoid biosynthesis in plants. In *Arabidopsis thaliana*, *AtMYB3*, *AtMYB4*, *AtMYB7*, and *AtMYB32* inhibit phenylpropanoid biosynthesis (Jin et al., 2000; Preston et al., 2004; Dubos et al., 2010; Fornalé et al., 2014). In *Salvia miltiorrhiza*, *SmMYB36* and *SmMYB39* prevent the accumulation of phenolic acid (Zhang et al., 2013; Ding et al., 2017). In *F. tataricum*, several MYB transcription factors were reported to activate or repress flavonoid biosynthesis. Overexpressing *FtMYB1* and *FtMYB2* enhances the biosynthesis and accumulation of anthocyanins (Bai et al., 2014). *FtMYB116* can be induced by red and blue light and promotes the accumulation of rutin by directly inducing the expression of flavonoid-3′-hydroxylase *(F3’H)*, which is involved in flavonoid biosynthesis (Zhang et al., 2018). The R2R3-MYB transcription factor *FtMYB6* is also induced by light and promotes flavonol biosynthesis by activating the expression of *FtF3H* and *FtFLS1* (Yao et al., 2020). *FtMYB11* represses phenylpropanoid biosynthesis (Zhou et al., 2017). *FtMYB13*, *FtMYB14*, *FtMYB15*, and *FtMYB16* are considered as negative regulators repressing rutin biosynthesis (Zhang et al., 2018).

The clustered regularly interspaced short palindromic repeat/CRISPR-associated protein 9 (CRISPR/Cas9) system has been recently developed from the adaptive immune system of *Streptococcus pyogenes* and is a powerful tool for targeted genome editing (Jinek et al., 2012). The CRISPR/Cas9 technology usually consists of two parts, an artificial single-guide RNA (sgRNA) and Cas9 nuclease. It has been successfully used for targeted gene modifications in a wide variety of plants (Feng et al., 2013), such as Arabidopsis (Jiang et al., 2014), rice (Shan et al., 2015; Srivastava et al., 2017), potato (Wang et al., 2015), maize (Shin et al., 2017), soybean (Cai et al., 2018), and rapeseed (Wang et al., 2017). However, the CRISPR/Cas9 system has been seldom used in *F. tataricum*. The polycistronic tRNA-sgRNA (PTG)/Cas9 system has been reported to be more efficient for gene editing in rice (Xie et al., 2015), kiwifruit (Wang et al., 2018), sweet orange (Tang et al., 2021), and grape (Ren et al., 2021). This technology uses the endogenous tRNA processing system to boost CRISPR/Cas9 gene editing capability. It consists of multiple tandemly arrayed tRNA-sgRNA units that form the PTG gene. Studies indicated that the start and end sites of the tRNA in the tandemly arrayed tRNA-sgRNA transcripts can be precisely recognized and cleaved by endogenous RNases (RNase P and RNase Z in plants) to simultaneously produce multiple functional sgRNAs (Xie et al., 2015).

Here, the PTG/Cas9 system was employed for targeted mutagenesis of *FtMYB45* in *F. tataricum*. The *FtMYB45 (MYB15)* gene has been identified as a transcriptional repressor of the flavonoid biosynthetic pathway, particularly of rutin (Zhang et al., 2018). In this study, the PTG/Cas9 system effectively induce mutations of the target gene *FtMYB45* in transgenic hairy roots, and the content of flavonoids such as rutin increased in mutant lines. Thus, the PTG/Cas9 gene editing system was efficacious in *F. tataricum*. To our knowledge, the present work is the first report of the CRISPR/Cas9 technology applied in *F. tataricum*, which provides a good technical foundation for molecular genetic studies in Tartary buckwheat.

## Materials and Methods

### Plant Materials and Growth Conditions

The Tartary buckwheat variety Jinqiao No. 2 used in this study was provided by Professor Qingfu Chen from Guizhou Normal University. The peeled seeds were soaked in distilled water for 20 min, then sterilized in 75% ethanol for 45 s and in 1 g L^−1^ mercuric chloride for 8 min, and washed 3–4 times with sterile water. Afterward, the seeds were blotted on filter paper to remove excess water and sown onto Murashige and Skoog (MS) medium in a greenhouse with a 16-h light/8-h dark photoperiod at 25°C.

For UV-B treatment, the seedlings were grown in full darkness for 5 days and then irradiated with UV-B light (300 nm, 2.0 × 100 μw/cm^2^) for 6 h. Seedlings kept in the dark were used as controls. The treatment comprised three biological replicates. Seedlings were frozen in liquid nitrogen and stored at −80°C.

### Quantitative Real-Time Reverse Transcription-Polymerase Chain Reaction

Total RNA was isolated using an RNA Extraction Kit (Takara, Dalian, Liaoning, China). First-strand cDNA synthesis was performed using 2 μg of the total RNA and PrimeScript™ RT reagent Kit (Takara), and qRT-PCR was conducted in a total volume of 20 μl on the qTOWER 3 real-time PCR system (ChemStudio SA, Analytik Jena, Germany) using SYBR Premix ExTaq Mix (Takara). The primers for qRT-PCR were designed using Primer Premier 5 (Premier Biosoft, United States) and are listed in Supplementary Table S1, and the Tartary buckwheat *Histone 3 (H3)* gene (GenBank accession number. JF769134) was used as an internal control gene (Gocal et al., 2001). PCR cycling began with a denaturing step at 95°C for 2 min, followed by 40 cycles at 95°C for 5 s, 60°C for 10 s, and 72°C for 10 s. Finally, the dissolution curve signals were collected from 60°C to 95°C. Three biological and three technical replicates were performed. The gene expression levels were analyzed using the 2^−ΔΔCT^ method (Livak and Schmittgen, 2001).

### sgRNA Design and Vector Construction

Two sgRNAs (sgRNA1 and sgRNA2) targeting *FtMYB45* were designed and their off-target effects were analyzed based on the published genome sequence of *F. tataricum* (GenBank accession number: GCA_002319775.1) and the website (Concordet and Haeussler, 2018).^1^ Secondary structure analysis of target-sgRNA sequences was carried out with the program RNA Folding Form (Ma et al., 2015b).^2^ The specific PCR primers C45-F and C45-R spanning sgRNA target sites were designed (Supplementary Table S1). A 360-bp fragment was amplified by PCR using genomic DNA of Jinqiao No. 2 as template, purified using a PCR purification kit (TransGen Biotech, Beijing, China), and sequenced to verify the sequences of sgRNA1 and sgRNA2.

The CRISPR/Cas9 vector targeting the *FtMYB45* gene was constructed using PTG/Cas9 method according to method of Wang et al. (2018). The sgRNA intermediate vector pHLW-sgRNA-tRNA and the Cas9 binary vector pPTG-sgRNA-Cas9-U6-1 were used. First, the fragment containing the first *Bsa* I site, sgRNA1, sgRNA scaffold, tRNA, sgRNA2, and the second *Bsa* I site was amplified from vector pHLW-sgRNA-tRNA using the target-specific primers 45sg-F and 45sg-R (Supplementary Table S1). Then, the PCR fragment was digested with restriction enzymes *Bsa* I (New England Biolabs, United States), and ligated into the BsaI-linearized vector pPTG-gRNA-Cas9-U6-1 with T4 DNA ligase (New England Biolabs, United States) to generate vector PTG/Cas9-FtMYB45. The ligation mixture was transformed into *Escherichia coli* DH5α competent cells and plated on LB-kanamycin agar plate (50 mg/L). Positive clones were confirmed by colony PCR using the primers SP-F and SP-R primers (Supplementary Table S1). All primers were synthesized commercially (Sangon Biotech Co., Ltd., Shanghai, China), *E. coli* competent cells were produced in our laboratory.

### *Agrobacterium rhizogenes*-Mediated Hairy Root Transformation in *Fagopyrum tataricum*

Hairy roots transformation of Tartary buckwheat mediated by *A. rhizogenes* was performed as previously reported (Mi et al., 2020). Briefly, the cotyledons and hypocotyls of 7–10 day-old old Tartary buckwheat seedlings were used as explants. The cotyledons were cut into small squares and the hypocotyls were cut into approximately 0.5-cm segments. All explants were precultured on MS solid medium for 1 day. The plasmid PTG/Cas9-FtMYB45 was introduced into *A. rhizogenes ACCC10060* by electrotransformation. *Agrobacterium rhizogenes ACCC10060* strain harboring PTG/Cas9-FtMYB45 was cultured in a shaker at 200 rpm and 28°C until the OD_600_ value reached 0.2. The prepared explants were soaked in the bacteria suspension for 10 min and cocultivated on a cocultivation medium (MS + 100mΜ acetosyringone) with filter paper at 25°C for 3 days in the dark. Then, the cocultured explants were transferred onto a selection medium (MS + 200 mg/L cefadroxil + 50 mg/L kanamycin) and cultured under a 16-h light/8-h dark cycle at 25°C for about 2 weeks until hairy roots were induced. Afterward, hairy roots were cut into 2–3 cm pieces and transferred into 100 ml glass bottles containing 10 ml of the selection medium and cultured in a shaker at 80 rpm at 25°C in the dark until they overspread to the bottom of the glass bottles (replace medium every 7 days if necessary). After 10–12 days, the hairy roots were collected and frozen at −80°C for identification and subsequent analysis.

### Determination of Flavonoid Metabolites by UPLC-QqQ/MS

Ground fresh hairy roots were accurately weighed and 0.1 g were extracted in 500 μl 70% methanol for 2 h at 4°C. The extract was sonicated for 30 min and centrifuged at 12,000 rpm for 10 min at 4°C, then filtered through a 0.22-μm hydrophilic organic nylon microporous membrane (SCAA-104). The extracted samples were analyzed by Agilent UPLC 1290II-G6400 QqQ MS (Agilent Technologies, Santa Clara, CA, United States) following the method published by Yang et al. (2020).

### Mutant Analysis

Genomic DNA was extracted from T0 transgenic hairy roots using the DNAsecure Plant Kit (TianGen Biotech Co., Ltd., Beijing, China). Positive transgenic hairy roots were verified by PCR, using primers specific from the kanamycin resistance gene (Kan-F and Kan-R; Supplementary Table S1). The primers C45-F and C45-R were used to amplify the sgRNA region. The PCR products were sequenced directly by C45-F and C45-R. The sequencing chromatograms were decoded using the Degenerate Sequence Decoding method (DSDecode) and predicted the mutant types (Liu et al., 2015; Ma et al., 2015a). To accurately identify the mutation types, the PCR fragment was purified and cloned into pEASY-T1 cloning vector (TransGen Biotech, Beijing, China), then the ligated product was identified by PCR and sequenced by Sanger sequencing. For each mutant line, at least 10 positive colonies were randomly selected and sequenced. The sequence alignment and mutation analysis were performed using the DNAMAN software (Version 4.0; Lynnon Corporation, Canada).

### Data Statistical Analysis

Student’s *t*-test and one-way analysis of the variance (ANOVA) were performed using GraphPad Prism8.0.1. *p* values <0.01 is considered statistically significant.

## Results

### *FtMYB45* Is Repressed by UV-B and Inhibits Flavonoid Biosynthesis

**Figure 1:**
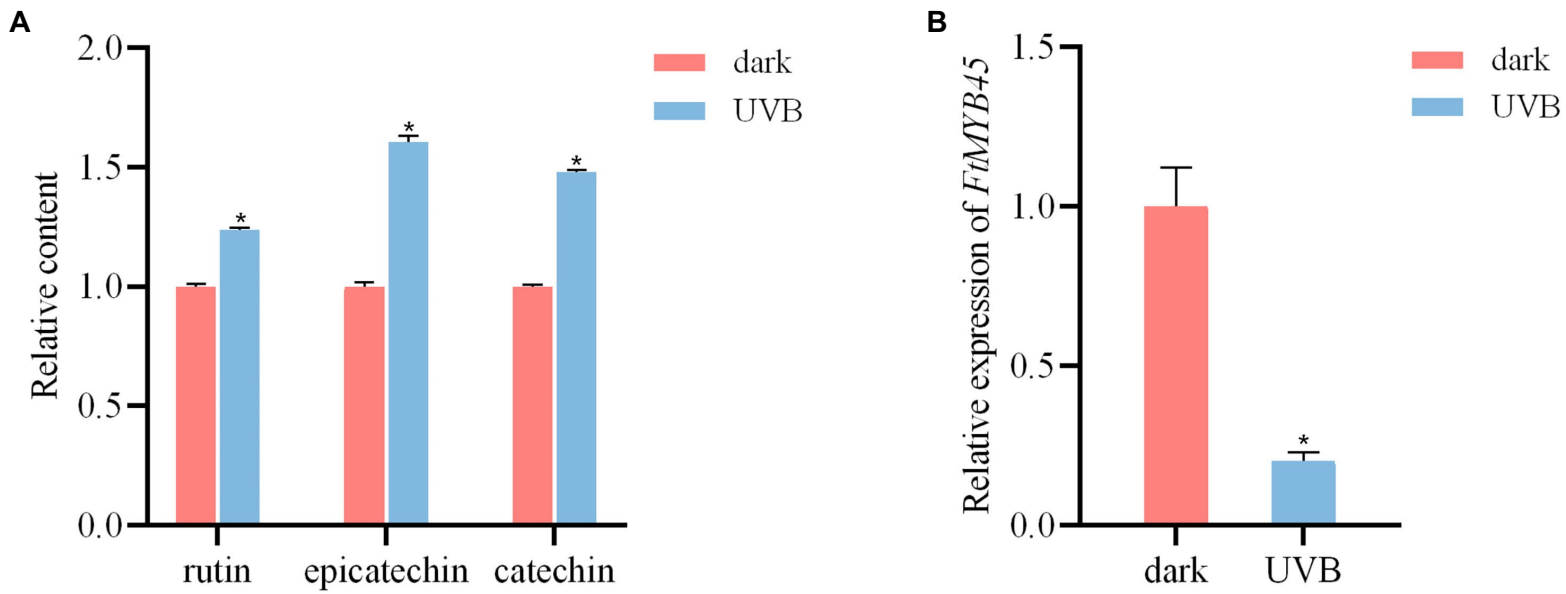
UV-B irradiation enhance the contents of flavonoids and reduce *FtMYB45* gene expression in Tartary buckwheat seedlings. **(A)** Content changes of rutin, epicatechin, and catechin in Tartary buckwheat seedlings after 6 h of UV-B treatment. **(B)** Changes of *FtMYB45* gene expression after 6 h of UV-B treatment. The values represent the means ± standard deviations (SDs) of three biological replicates. Asterisks indicate statistically significant differences compared with control seedlings under dark (^*^p < 0.01, Student’s *t*-test).

Ultraviolet-B (UV-B) is an important environmental signal that regulates plant growth and development. Previous studies have shown that UV-B can induce the key genes in the flavonoid biosynthetic pathway and increased the accumulation of flavonoids in *Ginkgo biloba* (Zhao et al., 2020), strawberry (Warner et al., 2021), apple (Hu et al., 2020), blueberry (Li et al., 2021), and other plant species (Suzuki et al., 2005; Huang et al., 2016). To investigate the effect of UV-B on the accumulation of flavonoids in Tartary buckwheat, 5-day-old seedlings were treated with UV-B light. Liquid chromatography–mass spectrometry (LC-MS) analyses showed that the content of rutin, epicatechin, and catechin were significantly increased after UV-B irradiation (*p* < 0.01; Figure 1A), indicating that UV-B irradiation promoted the accumulation of these flavonoids in Tartary buckwheat. Previous study revealed that the *FtMYB45* gene was induced by MeJA and repressed rutin biosynthesis (Zhang et al., 2018). Our qRT-PCR result indicated that the *FtMYB45* expression in seedlings was significantly downregulated (*p* < 0.01) after UV-B treatment compared with the control cultured in the dark (Figure 1B). It implied that *FtMYB45* was also repressed by UV-B treatment and inhibited flavonoid biosynthesis in Tartary buckwheat. Based on this result, *FtMYB45* was selected as the target gene for the development of a CRISPR/Cas9 workflow in Tartary buckwheat.

### SgRNA Design and PTG/Cas9-FtMYB45 Vector Construction

**Figure 2:**
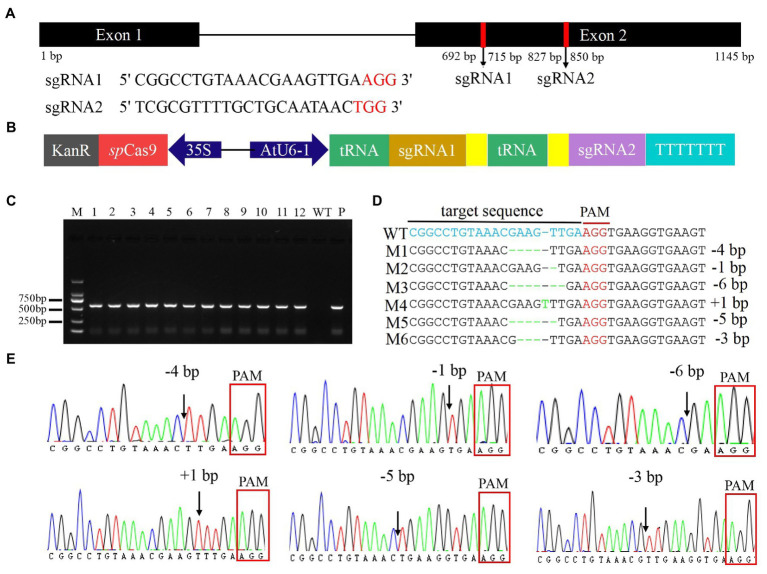
Construction of the PTG/Cas9-FtMYB45 vector and targeted modification of the *FtMYB45* gene. **(A)** Schematic illustration of the two sgRNAs target sites in the *FtMYB45* gene. The black rectangles represent exons, the black line represents the intron, and the numbers below represent the number of bases. The red vertical bars represent the locations of the sgRNA1 and sgRNA2. The red letters represent the protospacer adjacent motif (PAM) of each sgRNA. **(B)** Schematic diagram of the PTG/Cas9-FtMYB45 vector. The spCas9 expression cassette was driven by the CaMV 35S promoter, and the polycistronic tRNA-sgRNA cassette (PTG) was driven by the AtU6-1 promoter. The yellow rectangles represent the sgRNA scaffold. **(C)** Identification of the transgenic hairy root line by PCR amplification of the kanamycin resistance gene. The length of the PCR product was 563 bp. M represents the DNA marker DL2000. Lines 1–12 are individual hairy root lines, WT, wild type; P, positive control. **(D)** Mutation types induced by sgRNA1 in *FtMYB45*. The blue letters represent the sgRNA1 target sequence. The red letters represent the PAM sequence. The green letter represents the nucleotide insertion and the green dashes represent the nucleotide deletions. M1–M6 on the left side represent the mutation types. WT, wild type; +, insertion; −, deletion. **(E)** The sequencing chromatograms of mutation types of *FtMYB45*. The black arrowheads represent the locations of mutations. The red rectangles represent the PAM sequence.

The *FtMYB45* gene is located in chromosome 5 and is 1,145 bp in size, with two exons. Two sgRNAs targeting exon 2 of the *FtMYB45* gene were designed (Figure 2A), the GC content of sgRNA1 and sgRNA2 was 52.17 and 47.83%, respectively. On-target and off-target the designed sgRNAs were analyzed by the CRISPROR tool. The cutting frequency determination (CFD) score is widely used to measure sgRNA on-target specificity, and a high CFD specificity score indicates high sgRNA specificity (Doench et al., 2016). The result indicated the CFD score of sgRNA1 was 99 with 0 off-target within four mismatch bases, and the CFD score of sgRNA2 was 98 with only 1 off-target within four mismatch bases. Thus, both sgRNA1 and sgRNA2 were highly specific.

To verify the accuracy of the sgRNA sequences in Jinqiao No.2, the sgRNA region was amplified using the specific primer pair C45-F and C45-R and sequenced. The results showed that the sequences of sgRNA1 and sgRNA2 in Jinqiao No. 2 were 100% matched to the reference sequences (Supplementary Text S1). The PTG/Cas9-FtMYB45 vector was constructed according to the method published by Wang et al. (2018). In this vector, the spCas9 expression cassette was driven by the CaMV 35S promoter, and the polycistronic tRNA-sgRNA cassette (PTG) was driven by the AtU6-1 promoter (Figure 2B). After validation of the construct sequence by Sanger sequencing, the PTG/Cas9-FtMYB45 was introduced into *A. rhizogenes ACCC10060* cells for the transformation of *F. tataricum*.

### Targeted Mutagenesis of *FtMYB45* Gene Using the PTG/Cas9 System

Since the plant regeneration and genetic transformation have not yet been refined, *A. rhizogene*-mediated hairy root transformation is still the main method for genetic transformation in Tartary buckwheat. Our data indicated that *FtMYB45* was expressed in different organs of Tartary buckwheat, and was also expressed in hairy root (Supplementary Figure S1).

Therefore, *A. rhizogene* strain *ACCC10060* harboring the PTG/Cas9-FtMYB45 vector was transformed into Tartary buckwheat explants to induce hairy roots. Twelve transgenic hairy roots were obtained according to the PCR detection of the kanamycin resistance gene (neomycin phosphotransferase gene, nptII) using the primer pair Kan-F and Kan-R (Figure 2C). The sgRNA target region was amplified from the transgenic hairy roots using the specific primer pair C45-F and C45-R and sequenced to analyze *FtMYB45* mutations. The direct Sanger sequencing chromatograms were decoded by DSDecode (Supplementary Figure S2) and the mutation types were further genotyped by cloning and Sanger sequencing. The results showed that six hairy root lines (45–12, 45–13, 45–14, 45–17, 45–18, and 45–19) presented mutations at the target sites of sgRNA1 (Table 1; Supplementary Table S2), and the editing efficiency reached 50%. Sequence alignment revealed that there were six types of mutations (named M1–M6; Figure 2D), including insertion and deletion (Figure 2E). Among them, line 45–12 was a chimeric mutant, line 45–13, 45–14, 45–17, and 45–18 were biallelic mutants and line 45–19 was heterozygous mutant (Table 1). Unfortunately, no mutations were detected at the target sites of sgRNA2.

**Table 1:** Mutant genotypes and mutant type by Sanger sequencing analysis.

Mutant line	No. of clone sequenced	WT	Mutant type						Genotype
			M1 (−4 bp)	M2 (−1 bp)	M3 (−6 bp)	M4 (+1 bp)	M5 (−5 bp)	M6 (−3 bp)	
45–12	18		4	9	2	3			Chimeric
45–13	11			8		3			Biallele
45–14	11			9		2			Biallele
45–17	11					5	6		Biallele
45–18	11					6	5		Biallele
45–19	11	7						4	Heterozygote

*+, insertion; −, deletion; WT, wild type: reference sequences of sgRNA1 in the *FtMYB45* gene*.

### Changes in Flavonoids Content in *FtMYB45* Mutants

**Figure 3:**
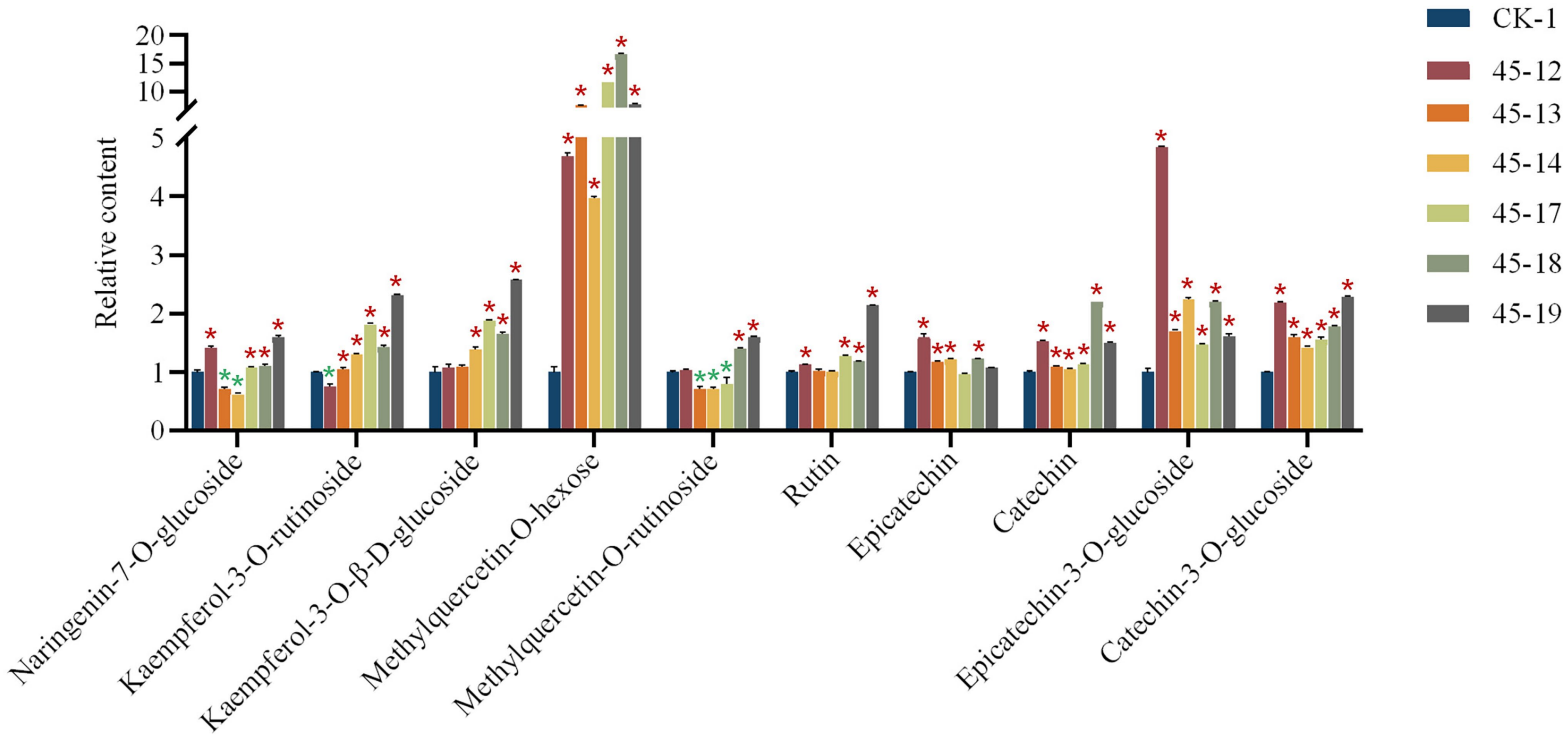
UPLC-QqQ-MS analysis of the changes of flavonoids content in mutant hairy root lines. CK represents hairy root transformed with the empty vector, 45–12, 45–13, 45–14, 45–17, 45–18, and 45–19 are individual mutant hairy root lines. The values represent the means ± standard deviations (SDs) of three biological replicates. Asterisks represent statistically significant differences compared with CK. The red asterisks represent significant increase, and the green asterisks represent significant decrease (^*^p < 0.01, one-way ANOVA).

UPLC-QqQ-MS metabolomics analysis (Yang et al., 2020) was used to determine the changes in flavonoids content in *FtMY*B45 mutant hairy roots compared with that in the control line. The main 10 flavonoids in hairy roots including naringenin-7-O-glucoside, kaempferol-3-O-rutinoside, kaempferol-3-O-β-D-glucoside, methylquercetin-O-hexose, methylquercetin-O-rutinoside, rutin, epicatechin, catechin, epicatechin-3-O-glucoside, and catechin-3-O-glucoside were detected (The chromatograms are shown in Supplementary Figure S3). The content of six flavonoids (kaempferol-3-O-β-D-glucoside, methylquercetin-O-hexose, rutin, catechin, epicatechin-3-O-glucoside, and catechin-3-O-glucoside) were increased in all *FtMYB45* mutant lines, and most of these increases were significant. However, the content changes of naringenin-7-O-glucoside, kaempferol-3-O-rutinoside, and methylquercetin-O-rutinoside in mutant lines were variable, for example, kaempferol-3-O-rutinoside in 45–12 line, naringenin-7-O-glucoside and methylquercetin-O-rutinoside in 45–13 and 45–14 lines, and methylquercetin-O-rutinoside in 45–17 line were significantly decreased, while they were increased in other mutant lines. Moreover, epicatechin levels were slightly diminished in the 45–17 and 45–19 (Figure 3). Altogether, the data indicated that *FtMYB45* mutation caused an increase of the amount of most flavonoids in hairy roots of Tartary buckwheat, suggesting that *FtMYB45* negatively regulated flavonoid biosynthesis.

## Discussion

Tartary buckwheat contains a large amount and variety of flavonoids, making it a popular health food. MYB transcription factors are key regulators of flavonoid biosynthesis in plants (Cao et al., 2020). Here, we focused on the flavonoids present in Tartary buckwheat, which, despite their importance, have not been thoroughly investigated. Previous studies demonstrated that *FtMYB45* was a JA responsive factor that repressed rutin biosynthesis (Zhang et al., 2018). Our study showed that UV-B irradiation significantly decreased the expression level of *FtMYB45* and significantly increased the content of rutin, epicatechin, and catechin in Tartary buckwheat (Figure 1), indicating that *FtMYB45* also inhibits flavonoid biosynthesis through UV-B signal transduction. Therefore, *FtMYB45* may be involved in the crosstalk between UV-B and JA signaling pathways, and regulate flavonoid biosynthesis in Tartary buckwheat. This finding provides new insight into the function of *FtMYB45* in Tartary buckwheat and might be of importance for the culture of Tartary buckwheat with high content in flavonoid metabolites.

The CRISPR/Cas9 gene editing is a fast, simple, efficient, and flexible technique for gene function analysis and crop improvement (Gupta et al., 2019; Triozzi et al., 2021). It has been widely used in a variety of plants (Jinek et al., 2012; Feng et al., 2013), and has also been applied in medicinal plants (Li et al., 2017; Feng et al., 2018, 2021). However, the application of CRISPR/Cas9 technology in Tartary buckwheat has not been reported yet. In this study, the *FtMYB45* was selected as the target gene to test the CRISPR/Cas9 system in Tartary buckwheat. Two sgRNAs of *FtMYB45* were designed to ensure efficient knockout of *FtMYB45*, and the off-target analysis indicated that sgRNA1 and sgRNA2 were highly specific. The PTG/Cas9-FtMYB45 vector was transformed into Tartary buckwheat using *A. rhizogenes* to induce transgenic hairy roots. Twelve transgenic hairy roots were obtained. Sequencing analyses showed successful gene editing in the region targeted by sgRNA1 in six hairy root lines, with the editing efficiency reaching 50%. A total of six types of mutations, including base insertions and deletions, were detected at the target site (Figure 2). Therefore, we successfully knocked out the *FtMYB45* gene in Tartary buckwheat using the PTG/Cas9 system, and consequently provide a new tool for gene function research and genetic improvement in Tartary buckwheat.

Unfortunately, no mutation was observed at the target sites of sgRNA2. The GC content of sgRNA has been considered as one of the key factors affecting sgRNA editing efficiency. Previous reports have shown that 97% of sgRNAs which have been experimentally validated in plants have a GC content between 30 and 80% (Liang et al., 2016). In our study, the GC content of sgRNA1 and sgRNA2 was 52.17 and 47.83%, respectively. Thus, GC content may not be the reason why sgRNA2 does not edit. Another main reason affecting sgRNA activity is the secondary structure of sgRNAs (Makarova et al., 2011). Assessment of the secondary structures of the studied sgRNA1 and sgRNA2 (Supplementary Figure S4) found that sgRNA2 formed an 8 bp typical stem-loop structure. Ma et al. (2015b) also reported an inactive sgRNA formed a stem-loop structure with a pairing of continuous 14 and 4 bp of the target, and suggested the sgRNA selection should avoid those with pairing to the sgRNA by more than continuous 6 bp. Thus, the continuous 8 bp stem-loop structure might inhibit the binding of the sgRNA2 to the target strand, leading to the failure of gene editing.

Hairy root cultures established by transforming plants with *A. rhizogenes* have been utilized to produce transgenic plants, investigate plant metabolic processes, and increase secondary metabolites. They are genetically and biochemically stable during rapid growth (Guillon et al., 2006a,b). Recently, the hairy root transformation has been widely utilized to validate and optimize induced mutagenesis by the CRISPR/Cas9 system (Li et al., 2019; Le et al., 2020). In addition, biotechnological approaches which used hairy root culture have greatly enhanced the production of rutin by common buckwheat (Lee et al., 2007; Kim et al., 2010). Therefore, hairy root cultures have been used as a useful model system to study the production of flavonoids and a variety of other secondary metabolites. Now, the flavonoid biosynthesis pathway is relatively clear (Supplementary Figure S5; Falcone Ferreyra et al., 2012; Dong and Lin, 2021). Dihydroflavonols are precursors used for flavonoid biosynthesis. Flavonol synthase (FLS) links flavonoids and flavonols synthesis pathways and is involved in dihydroflavonol desaturation to form flavonols (Forkmann and Martens, 2001). Dihydroflavonol reductase (DFR) is a key enzyme and an important branch point in the synthesis pathway of anthocyanins and catechins (Landry et al., 1995). In this study, we detected 10 flavonoids including flavonols, flavanols, and their glycosides in the obtained *FtMYB45* mutant hairy root lines. The UPLC-QqQ-MS result showed that the content of most of these flavonoids was significantly increased in mutant lines. In particular, the content of methylquercetin-O-hexose, proanthocyanidins, including catechin, epicatechin-3-O-glucoside, and catechin-3-O-glucoside, were greatly increased in all six mutant lines. Thus, our data demonstrated that *FtMYB45* is a negative regulator of flavonoid biosynthesis. This is consistent with the previous report showing that *FtMYB45* directly represses phenylalanine ammonia-lyase (*FtPAL*) gene expression, and thus affecting the entire flavonoid metabolic pathway (Zhang et al., 2018). Moreover, flavonoids content among the six mutant lines showed different change levels, which may be due to different mutations types of *FtMYB45*. The mutant lines with the same genotype showed similar content changes in detected flavonoids, for example, line 45–13 and 45–14, line 45–17, and 45–18 (Figure 3). However, the increase of some flavonoids in heterozygote mutant line 45–19 was greater than that of biallelic or chimeric mutant lines, which does not meet our expectations. The possible reason we suppose is that the Transfer DNA (T-DNA) insertion in line 45–19 may affect the related genes in flavonoid biosynthesis. We also noticed that the content of some flavonoids showed decreased in few mutant lines, for example, kaempferol-3-O-rutinoside in line 45–12, and naringenin-7-O-glucoside in line 45–13 and 45–14. The reason is still not clear and needs to be further studied.

Taken together, our results indicated that the application of the PTG/Cas9 gene editing system effectively knocked out *FtMYB45* and increased the content of flavonoids in mutant hairy roots in Tartary buckwheat. These *FtMYB45* mutant hairy root lines will be good candidate biomaterials for the production of flavonoids.

## Conclusion

In this study, the PTG/Cas9 genome editing system was successfully utilized for genome editing in Tartary buckwheat, which lays a valuable foundation for the application of CRISPR/Cas9 technology in gene function study and molecular breeding in Tartary buckwheat. Additionally, we performed targeted mutagenesis of the *FtMYB45* gene, which resulted in an increased content of flavonoids in mutant hairy roots of Tartary buckwheat. This finding provides further evidence to support the negative regulatory role of the *FtMYB45* gene in the flavonoid biosynthetic pathway, and the obtained mutant hairy root lines with increased amounts of flavonoids will provide good sources for the production of flavonoids.

## Data Availability Statement

The original contributions presented in the study are included in the article/**Supplementary Material**, and further inquiries can be directed to the corresponding authors.

## Author Contributions

DW performed the experiments, analyzed the data, and wrote the paper. MW and XW performed part of the hairy root transformation experiment. LW and WY analyzed part of the data. WM, WS, and SC revised the paper. YS and LX initiated and supervised the project. All authors contributed to the article and approved the submitted version.

## Funding

This study was supported by the CACMS Innovation Fund (CI2021A04107), the Fundamental Research Funds for the Central Public Welfare Research Institutes (ZZ13-YQ-101), National Key R&D Program of China from the Ministry of Science and Technology of China (2019YFC1711100), and the Agilent Thought Leader Program and ACT-UR Program.

## Conflict of Interest

The authors declare that the research was conducted in the absence of any commercial or financial relationships that could be construed as a potential conflict of interest.

## Publisher’s Note

All claims expressed in this article are solely those of the authors and do not necessarily represent those of their affiliated organizations, or those of the publisher, the editors and the reviewers. Any product that may be evaluated in this article, or claim that may be made by its manufacturer, is not guaranteed or endorsed by the publisher.

## Footnotes

^1^ http://crispor.tefor.net/

^2^ http://mfold.rna.albany.edu/?q=mfold/RNA-Folding-Form2.3
