# Supplementary material for: CRISPR/Cas9-Mediated Targeted Mutagenesis of FtMYB45 Promotes Flavonoid Biosynthesis in Tartary Buckwheat (Fagopyrum tataricum)
Source: Front Plant Sci. 2022 May 12;13:879390. doi: 10.3389/fpls.2022.879390 (PMC9133938; doi:10.3389/fpls.2022.879390)
Supplement: Supplementary file 1 [file Data_Sheet_1.docx]

Supplementary Material

# Supplementary Figures

##
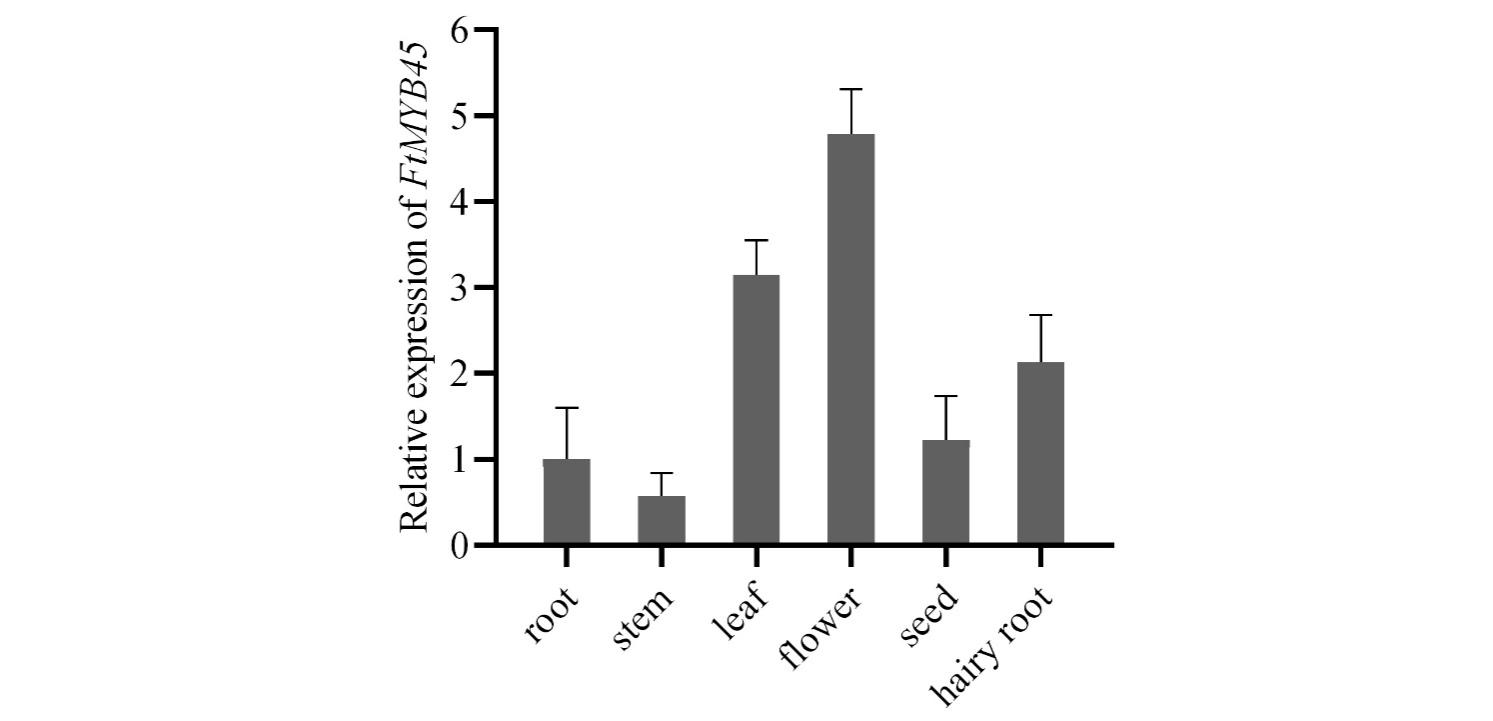


**Supplementary Figure S1** Tissue-specific gene expression of the *FtMYB45* gene in Tartary buckwheat. The root, stem, leaf, and flower were sampled from two-month-old plants, and the seed was sampled from three-month-old plants. Hairy roots were induced from the hypocotyls and cotyledons of Tartary buckwheat using *Agrobacterium rhizogenes-*mediated transformation. The values represent the means ± standard deviations (SDs) of three biological replicates.


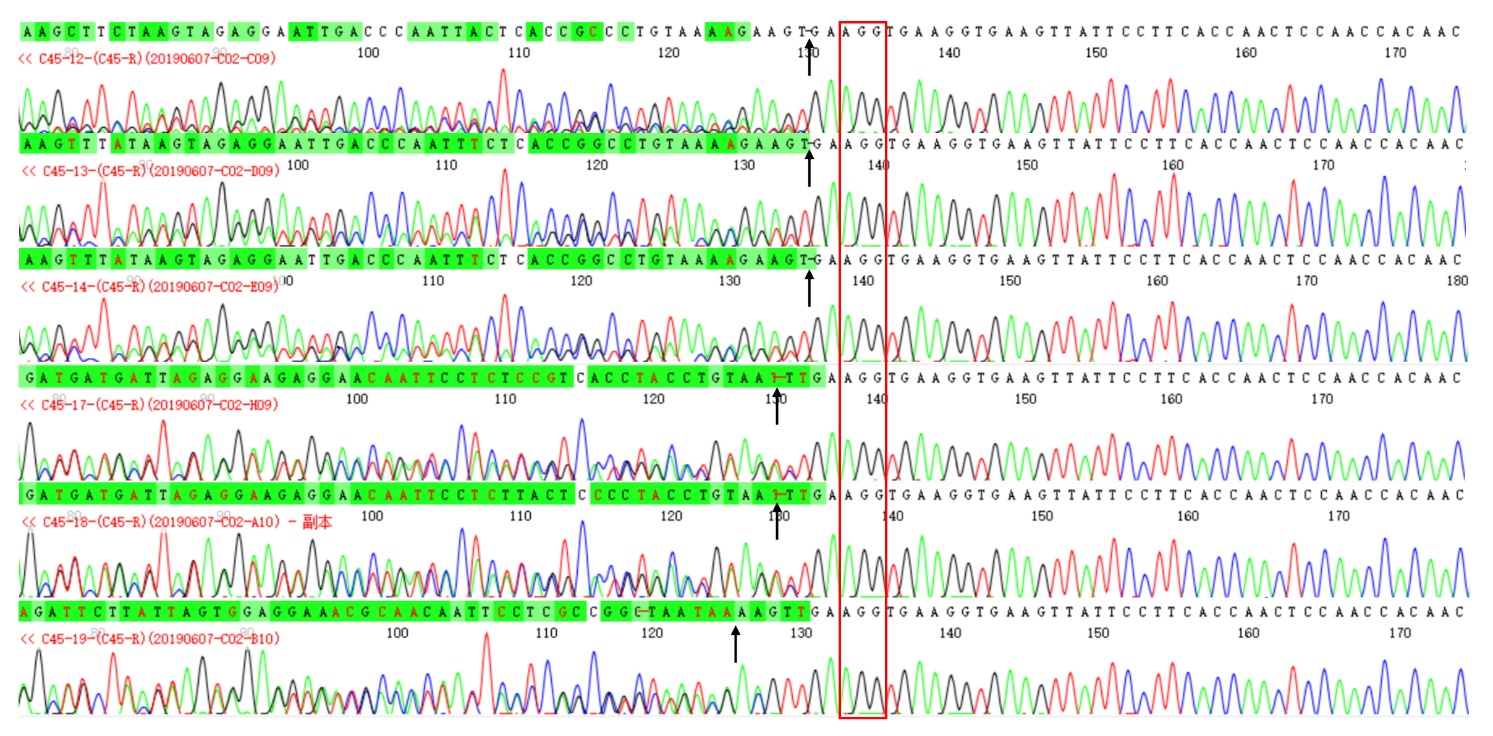


**Supplementary Figure S2** DNA sequence chromatograms of the *FtMYB45* gene from transgenic hairy roots. The red rectangle represents the PAM sequences, and the black arrows represent the DSB cleavage sites (3 bp upstream of the PAM sequences).


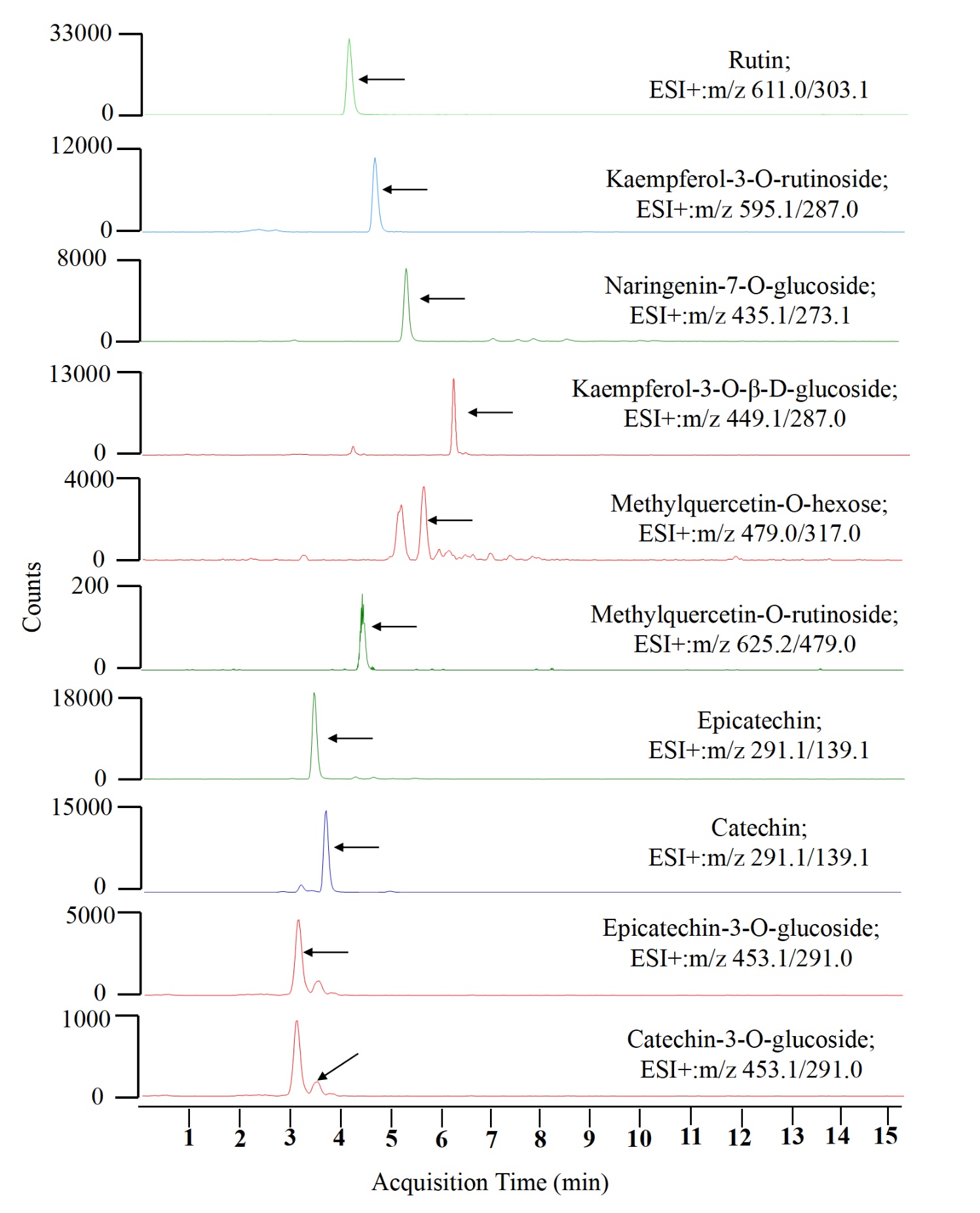


**Supplementary Figure S3** Extracted ion chromatograms of ten flavonoids detected in hairy roots of Tartary buckwheat. The compounds were analyzed in the positive ion mode (Electron Spray Ionization, ESI+).


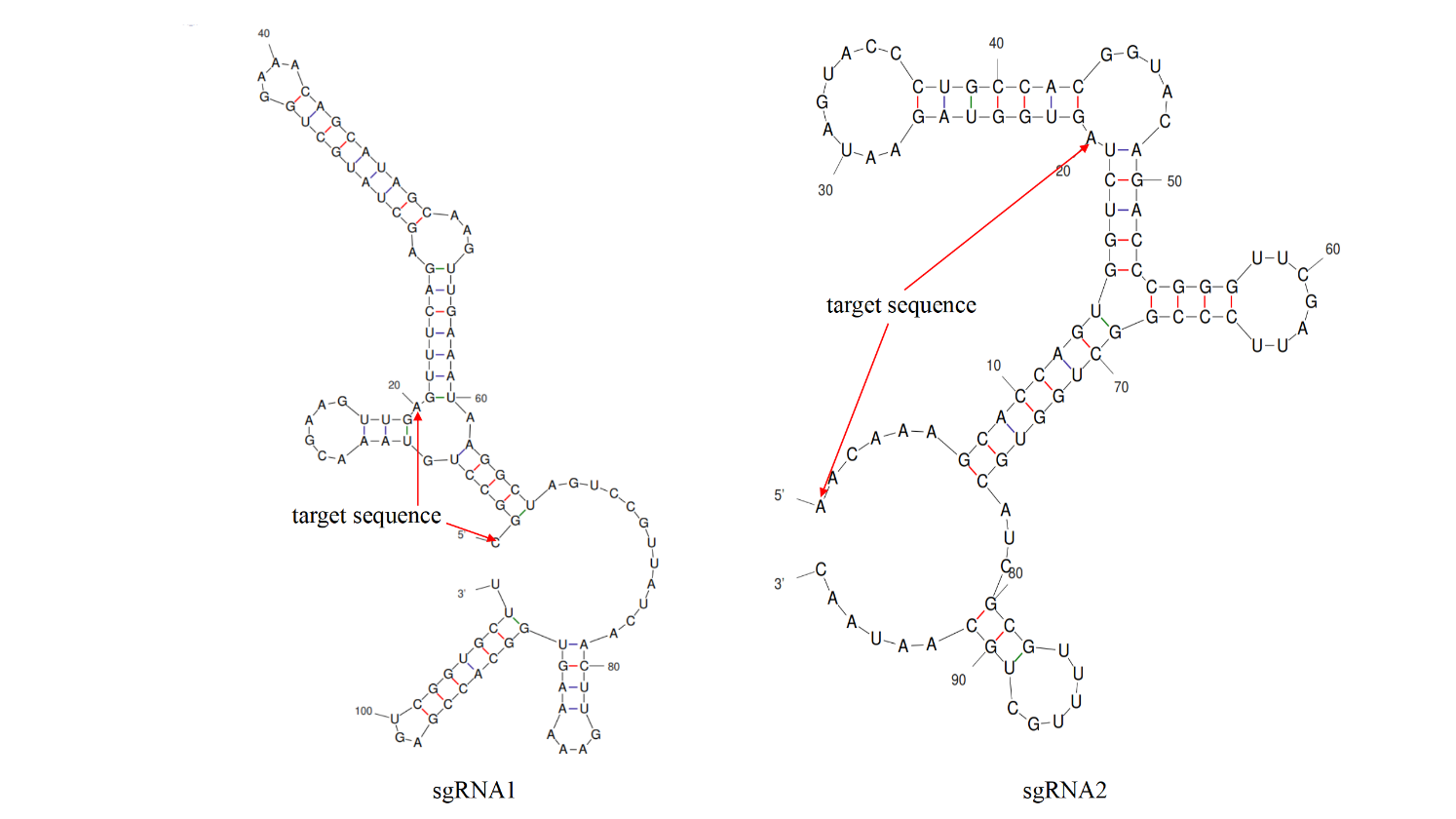


**Supplementary Figure S4** The secondary structures of sgRNA1 and sgRNA2 were analyzed using the program RNA Folding Form. (<http://mfold.rna.albany.edu/?q=mfold/RNA-Folding-Form2.3>).

Target-sgRNA:

(sgRNA1)CGGCCTGTAAACGAAGTTGAGTTTCAGAGCTATGCTGGAAACAGCATAGCAAGTTGAAATAAGGCTAGTCCGTTATCAACTTGAAAAAGTGGCACCGAGTCGGTGCTT

(sgRNA2)AACAAAGCACCAGTGGTCTAGTGGTAGAATAGTACCCTGCCACGGTACAGACCCGGGTTCGATTCCCGGCTGGTGCATCGCGTTTTGCTGCAATAAC


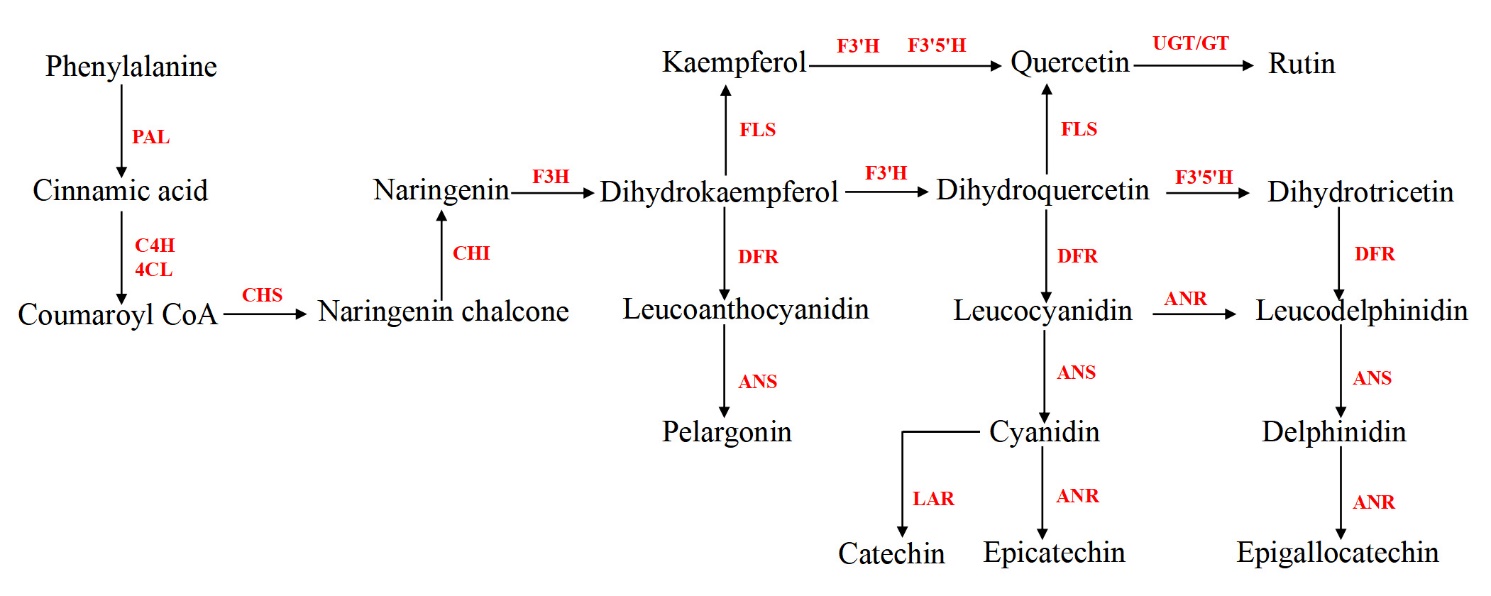


**Supplementary Figure S5** Flavonoid biosynthetic pathway in Tartary buckwheat. PAL: phenylalanine ammonia-lyase; C4H: cinnamate-4-hydroxylase; 4CL: 4-coumarate coenzyme A ligase; CHS: chalcone synthase; CHI: chalcone isomerase; F3H: flavanone-3-hydroxylase; FLS: flavonol synthase; F3'H: flavonoid-3'-hydroxylase; F3'5'H: flavonoid-3',5-'hydroxylase; DFR: dihydroflavonol-4-reductase; ANS: anthocyanidin synthase; ANR: anthocyanidin reductase; LAR: leucoanthocyanidin reductase; UGT/GT: glucosyl/rhamnosyl transferase.

**Supplementary Tables**

**Supplementary Table S1** Primer sequences used in this study.

| **Primer Name** | **Primer Sequence (5'-3')** | **Experiment** |
| --- | --- | --- |
| qFtMYB45-F | AGATTACCGGGGAGAACCGA | qRT-PCR of *FtMYB45* gene |
| qFtMYB45-R | ACAGGCCGGTGAGTAATTGG |  |
| FtH3-F | AAGGAAGCAATTGGCAAC | qRT-PCR of the *Histone 3* gene |
| FtH3-R | TCACGAAGAGCAACGGTA |  |
| C45-F | ATGGTCTTTGATTGCGGGAA | genotyping analysis |
| C45-R | TGATGTTGATGAGAAGGAG |  |
| 45sg-F | **GGTCTC**TTGCACGGCCTGTAAACGAAGTTGAGTTTCAGAGCTATGCTGGA | vector construction for PTG/Cas9-FtMYB45 |
| 45sg-R | **GGTCTC**TAAACTCGCGTTTTGCTGCAATAACTGCACCAGCCGGGAATCGA |  |
| SP-F | GTCGTGCTCCACATGTTGACCGG | colony PCR |
| SP-R | CCCGACATAGATGCAATAACTTC |  |
| KanR-F | ATTCGGCTATGACTGGGCAC | transgenic line identification |
| KanR-R | TGAATCCAGAAAAGCGGCCA |  |

The bold letters indicate the BsaI restriction sites (5'-GGTCTCN|NNNN-3', N indicates any nucleotide). The blue letters represent the sgRNA sequences. The underlined sequences indicate the gRNA scaffold homology arm and tRNA homology arm sequence of intermediate vector pHLW-sgRNA-tRNA, respectively.

**Supplementary Table S2** Mutations of the *FtMYB45* gene induced by PTG/Cas9 system at the sgRNA1 target site.

| **Line** | **Target Sequence** | **Gene Editing** | **Mutation Type** |
| --- | --- | --- | --- |
| sgRNA1 | CGGCCTGTAAACGAAGTTGAAGGTGAAGGTGAAGT | - | - |
| 45-12 | CGGCCTGTAAAC----TTGAAGGTGAAGGTGAAGT | -4 bp | M1 |
|  | CGGCCTGTAAACGAAG-TGAAGGTGAAGGTGAAGT | -1 bp | M2 |
|  | CGGCCTGTAAAC------GAAGGTGAAGGTGAAGT | -6 bp | M3 |
|  | CGGCCTGTAAACGAAGTTTGAAGGTGAAGGTGAAGT | +1 bp | M4 |
| 45-13 | CGGCCTGTAAACGAAG-TGAAGGTGAAGGTGAAGT | -1 bp | M2 |
|  | CGGCCTGTAAACGAAGTTTGAAGGTGAAGGTGAAGT | +1 bp | M4 |
| 45-14 | CGGCCTGTAAACGAAG-TGAAGGTGAAGGTGAAGT | -1 bp | M2 |
|  | CGGCCTGTAAACGAAGTTTGAAGGTGAAGGTGAAGT | +1 bp | M4 |
| 45-17 | CGGCCTGTAAACGAAGTTTGAAGGTGAAGGTGAAGT | +1 bp | M4 |
|  | CGGCCTGTAAAC-----TGAAGGTGAAGGTGAAGT | -5 bp | M5 |
| 45-18 | CGGCCTGTAAACGAAGTTTGAAGGTGAAGGTGAAGT | +1 bp | M4 |
|  | CGGCCTGTAAAC-----TGAAGGTGAAGGTGAAGT | -5 bp | M5 |
| 45-19 | CGGCCTGTAAACGAAGTTGAAGGTGAAGGTGAAGT | WT | - |
|  | CGGCCTGTAAACG---TTGAAGGTGAAGGTGAAGT | -3 bp | M6 |

The blue letters represent the target sequence of sgRNA1. The red letters represent the PAM sequence. The green letters represent the nucleotide insertions, and green dashes represent the nucleotide deletions. +, insertion; -, deletion; WT, wild-type.

**Supplementary Text**

**Supplementary Text S1** The coding sequence of *FtMYB45* gene

ATGGGTCGATCTCCATGTTGCGAGAAAGCTCATACAAACAAAGGTGCATGGACTAAAGAAGAAGATGATCGGTTAATTGCTTATATAAAAACTCACGGTGAAGGCTGTTGGAGATCTCTTCCTAAAGCTGCTGGACTTCTCCGATGTGGGAAGAGTTGCCGTCTCCGGTGGATTAACTATCTCCGACCTGATCTTAAGCGAGGAAACTTCACCGAGGAAGAAGATGAACTCATCATCAAGTTGCATAGCCTTCTTGGCAACAAGTTAGTTTCTTTTCCTCTTCCTTTGTTTCTATTGGTTTGTTTGAACAGGAAAGAAAACTATAACCTTTGACCAATGATTGCAAGAAAGATCGGGTCTTTAGAAATAAAATATGAGTCCATATTGGACATTGGAACCTTAGAAGATTTTGAAAAAGGTAAAAAGGAACAAAAAATTTTCTTTTGACTTTTTGCAAGATCCGTTCTTTGAACTAAAAAAGAAAAATGGGTGATGATCTTATTTATGCGATTAGGAATATGATTCATGAATTTAAAAGATATTGGACTGATCAAACTCAACCATTAATTATGTGCAGATGGTCTTTGATTGCGGGAAGATTACCGGGGAGAACCGATAATGAAATAAAAAACTACTGGAATACTCACATAAAGAGGAAGCTTCTAAGTAGAGGAATTGACCCAATTACTCACCGGCCTGTAAACGAAGTTGAAGGTGAAGGTGAAGTTATTCCTTCACCAACTCCAACCACAACAACTTCCATTTCATTTGGTGCTACTATACCTAAACTAGAGCAAGATTTACATGATCACAATCTCATGACGAATTCGCGTTTTGCTGCAATAACTGGATCCAAATCGGAAGAGAGGAATCCGGGTTATCGATGCCCGGATTTGAACTTGGAGTTAAGAATCAGCCCTCCTTCTCATCAACATCAGCCAGAACAACTGAAGAGTGGGGGCATAATTGTCAATTCCAACCCTTGCTTTAAATGTAGTTTTGGTATGCAAAGTGGCCAAGTTTGCAAGTGTAACACATTTGATAGCAAAGGTAATAGTAGTGAGTGTAGCAACATTGGCTTCCATGACTTCTTGGGGTTGAGAGGAAGTGCTACTTTGGACTATAGAACTTTGGAGATGAAATGA

The blue letters represent the sgRNA1 and sgRNA2. The red letters represent the PAM sequences.
